# Research progress of orbital fat in histology and cytology: A review

**DOI:** 10.1097/MD.0000000000039040

**Published:** 2024-07-26

**Authors:** Changhao Li, Shenzhen Gao, Weicheng Gao

**Affiliations:** a Department of Plastic and Cosmetic Surgery, The Affiliated Friendship Plastic Surgery Hospital of Nanjing Medical University, Nanjing, Jiangsu, China.

**Keywords:** anatomy, embryology, histology, orbital fat, orbital fat-derived stem cells

## Abstract

Orbital fat is an adipose tissue located behind orbital septum and originates from mesoderm and neural crest in ectoderm. It has been found that the histologic structure of orbital fat is different from subcutaneous and visceral fat. In addition, the regeneration and anti-inflammatory ability of stem cells derived from orbital fat have attracted much attention in recent years. This paper reviews the recent research progress on orbital fat, including its structure, origin, histological characteristics, and related stem cells.

## 1. Introduction

Adipose tissue had long been regarded as a metabolically inert tissue that stores excess energy, but with in-depth research in the past 30 years, we have found that adipose tissue has complex and diverse functions.^[1]^ The research progress in endocrine and immune aspects of adipose tissue provides new breakthroughs in the pathogenesis and treatment of many diseases, and the application of adipose stem cells in the regeneration field is also constantly making new progress. It is found that adipose tissue in different regions may have different physiological functions.^[2]^ For example, epicardial fat affects myocardial function through paracrine, and omental fat participates in the immune process through the milky spots. In recent years, related studies have shown that orbital fat may affect the function of surrounding tissues such as levator aponeurosis, orbital fat-derived stem cells have stronger multi-directional differentiation and anti-inflammatory ability than subcutaneous fat-derived stem cells, and orbital fat loss is also one of the reasons for the complications of blepharoplasty, such as sunken upper eyelid and high eyelid folds, so more in-depth research on orbital fat will help better guide clinical work. This review mainly reveals the potential physiological functions and great potential of clinical application of orbital fat by comparing the histological and cytological differences between orbital fat and subcutaneous fat.

## 2. Anatomical study of orbital fat

Orbital fat, also known as adipose body of the orbit, located behind the orbital septum, fills between the eyeball, muscles, blood vessels, and nerves. The fat located between the extraocular muscles and the eyeball is called central fat and consists of large lobules and thin connective tissue septa; the fat located between the extraocular muscles and the orbital wall is called peripheral fat and consists of smaller lobules and thick connective tissue septa.^[3]^ Due to its special location and complex shape, orbital fat has continued to make progress in its anatomical structure. Fan Hao et al^[4]^ showed that peripheral fat and central fat communicate through muscle gap; peripheral orbital fat is divided into anterior aponeurosis and posterior aponeurosis according to their distribution, and upper and lower eyelids are demarcated by levator aponeurosis and capsulopalpebral fascia respectively; diaphragm tissue exists between fat masses in anterior aponeurosis of peripheral orbital fat, which are not communicated with each other, but fat masses in posterior aponeurosis fuse with each other; there is no clear communication between the anterior aponeurosis of the upper and lower peripheral orbital fat at the attachment of the medial and lateral canthus tendons, but there is mutual communication after the insertion of the medial canthus tendon; the anterior aponeurosis of the peripheral orbital fat extends posteriorly through the passage between the muscle and the periosteum, and is a continuous whole. According to the study of Kakizaki et al,^[5]^ the orbital fat of the upper eyelid is generally composed of central or preaponeurotic fat pad and medial or nasal fat pad, but there is no such boundary in the deep part of the orbit; the central fat pad lies in front of the levator aponeurosis and is dark yellow or bright yellow in color, laterally it extends behind the lacrimal gland, and medially it is separated from the medial fat pad by the reflex tendon of the superior oblique muscle and the medial angle of the levator aponeurosis. The preaponeurotic fat pad was initially thought to be isolated from the orbital fat mass,^[6]^ but later studies suggested that they were a continuous whole.^[3]^ As mentioned above, orbital fat of upper and lower eyelids is a continuous whole, and less orbital fat of lower eyelid will affect orbital fat of upper eyelid, thus affecting eyelid appearance.^[7]^ Loss of orbital fat is also one of the reasons for complications after blepharoplasty, such as sunken upper eyelid and high eyelid folds. Therefore, when performing blepharoplasty involving removal of orbital fat or displacement, it is necessary to comprehensively consider clinical effects and carefully handle.

## 3. Embryological study of orbital fat

Since Walther Flemming discovered that some adipose tissue originated from connective tissue derived from mesoderm in the 1870s, adipose tissue had been thought to originate from mesoderm due to lack of embryological studies on adipose tissue.^[8]^ However, Billon et al^[9]^ found that retroauricular fat in mouse originated from neural crest of ectoderm by lineage tracing with cre recombinase in 2007, indicating that adipose tissue has different embryological origin. Orbital fat has received widespread attention as an easily available research material because it is often treated in cosmetic surgery of the upper and lower eyelids. Combined with the fact that orbital fat has different morphological characteristics from subcutaneous fat, researchers speculate that it has a different embryological origin. Johnston et al^[10]^ found that orbit and its contents originated from mesoderm and ectoderm through avian embryology research, among which corneal endothelial cells and orbital connective tissue originated from neural crest of ectoderm. Korn et al^[11]^ found that adipose stem cells extracted from medial fat pad of orbital fat expressed CD34 twice as much as that of preaponeurotic fat at the same period, while the low expression amount of central fat pad was similar to that of stem cells reported from subcutaneous fat. Reali et al^[12]^ suggested that CD34 is related to the ability of hematopoietic stem cells to differentiate into neural cell lineage. Therefore, CD34 may be a marker for the differentiation of stem cells into neural cells. Cells originating from neural crest of ectoderm often have the ability to differentiate into nerve cells.^[13]^ Therefore, we can boldly speculate that the medial fat pad originates from neural crest that are different from the central fat pad. However, in the experiment of differentiation of orbital fat-derived stem cells into neural cells in vitro, stem cells from central fat pad expressed more marker antigens of neuronal and glial cell^[11]^; while Sanie-Jahromi et al^[14]^ pointed out that compared with central fat pad, adipose stem cells from medial fat pad have stronger neural differentiation ability. Gola et al^[15]^ reported that medial fat pad of orbital fat is rich in fiber and blood vessels and is lobulated, while the central fat pad has less blood vessels and is liquid, similar to subcutaneous fat; Kim et al^[16]^ studied the development of upper eyelid of human embryo and found that the central fat pad of upper eyelid began to differentiate at 16 weeks, and the preseptal fat located between orbicularis oculi muscle and orbital septum began to differentiate at 18 weeks, which may indicate that the origin of the above fat is different, but failed to find the difference in differentiation time between medial and central fat pad. In summary, it is considered that the central and medial fat pad of upper eyelid originates from mesoderm and ectoderm respectively, but more clear evidence is still needed.

## 4. Histological characteristics of orbital fat

It is found that orbital fat is different from subcutaneous fat and visceral fat in histologic structure and cellular composition. Afanas et al^[17]^ showed that compared with subcutaneous fat and visceral fat, the adipocytes of orbital fat arranged more closely, the cells were smaller and the number of macrophages was more; the expression levels of FABP4 and G3PDHmRNA were lower, and FABP4 and G3PDH were the main markers of late adipocyte differentiation, indicating that the differentiation degree of adipocytes in orbital fat was lower. Using microscopic cameras to photograph sections and image processing software to process the data, we will obtain accurate physical data of orbital fat and subcutaneous fat. Bujalska et al^[18]^ found that the average diameter and volume density of adipocytes in orbital fat were much lower than those in subcutaneous fat, but the volume density of blood vessels and connective tissue in orbital adipose tissue was significantly higher than that in subcutaneous fat. In conclusion, the tissue structure of orbital fat was different from that of subcutaneous fat, so the method of using subcutaneous fat transplantation to fill eyelid depression may be questionable. However, the histological structure and cellular composition of orbital fat differs from subcutaneous fat in producing those functional differences that require further study.

The difference between orbital fat and subcutaneous fat is not only reflected in the histological structure, but also different in carotenoids and other components. Kaili Zhang et al^[19]^ found that the carotenoid content of orbital septum fat is significantly lower than that of subcutaneous fat, while the carotenoid content of central fat pad is significantly higher than that of medial fat pad,^[20]^ which is also the main reason for the color difference of orbital fat in different regions. Ahmadi et al^[21]^ found a tendency to decrease carotenoid content in the central fat pad of patients with degenerative ptosis compared to the normal population through case–control studies. Carotenoids have antioxidant and anti-inflammatory effects,^[22]^ and recent epidemiological studies have shown that carotenoids or carotenoid-rich foods can prevent muscle strength decline and walking disorders of the elderly in the community.^[23]^ In conclusion, carotenoids in the central cellulite may have a protective effect on the levator aponeurosis and levator muscle of upper eyelid as adjacent tissues.

## 5. Orbital fibroblasts

Intraorbital soft tissue enlargement is the main pathological change of Graves ophthalmopathy (GO). Most patients have enlargement of extraocular muscle and orbital adipose tissue at the same time, and some patients have one of them. However, the exact mechanism of intraorbital soft tissue enlargement in GO is unclear. In recent years, it is considered that orbital fibroblasts of patients with GO (GO-Fs) are important target cells.^[24]^ TERRY et al^[25]^ found that GO-Fs can differentiate into adipocytes in vitro culture by flow cytometry and directed differentiation medium, while fibroblasts from extraocular muscles cannot be induced to differentiate into adipocytes, indicating that the transformation of orbital fibroblasts into adipocytes may be one of the pathogenesis of GO. Katarzyna et al^[26]^ also pointed out that GO-Fs can be induced to differentiate into different types of cells such as fat, bone, cartilage, and nerve in vitro, and express surface antigens similar to mesenchymal stem cells. However, the results of SvenBrandau et al^[27]^ showed that compared with stem cells derived from orbital fat, GO-Fs had similar morphology, surface antigen and multi-differentiation ability, but they had obvious differences in differentiation potential, T cell inhibition and cytokine release. In conclusion, fibroblasts derived from orbital septum fat had great pathological changes in graves ophthalmopathy, and further study of the molecular mechanism of their phenotypic changes would help to deepen the understanding of the occurrence and development process of the disease.

## 6. Orbital fat-derived stem cells (OFSCs)

The pluripotent stem cells derived from orbital fat were first isolated and identified by Bobby et al^[11]^ in 2009, and can differentiate into adipocytes, smooth muscle cells, neurons, and glial cells, and express surface antigens consistent with those of adipose stem cells from other fat depots. Chen et al^[28]^ showed that OFSCs have the pluripotent differentiation ability to differentiate into adipocytes, osteocytes and chondrocytes; direct contact between OFSCs and corneal epithelium can induce it to differentiate into corneal epithelial cells to repair damaged corneas, while adipose stem cells derived from subcutaneous fat and mesenchymal stem cells derived from bone marrow are difficult to differentiate into corneal epithelial cells under the same culture conditions.

Martins et al^[29]^ also found that stem cells could not differentiate into corneal cells when OFSCs were transplanted into decellularized corneal stroma, which indicated that direct contact with corneal epithelial cells was a necessary condition for inducing OFSCs to differentiate into corneal epithelial cells. Some scholars^[30,31]^ found that animal models with corneal damage can be recovered to some extent by local instillation of OFSCs mixture or injection of regenerated corneal epithelial cells after mixed culture of OFSCs and corneal epithelial cells in vitro into damaged cornea. However, local administration often requires multiple administrations and the efficacy is difficult to control, the difficulty in extraction and purification of regenerated corneal epithelial cells after mixed culture limits clinical application. Chien et al^[32]^ showed that OFSCs have the same low immunogenicity as mesenchymal stem cells derived from bone marrow, so it is possible to transplant allogeneic OFSCs to regenerate corneal cells. Some studies showed that OFSCs can repair nerve damage and retinal degenerative damage through their multi-directional differentiation ability.^[33,34]^ In conclusion, OFSCs have extensive and far-reaching application prospects in the field of ocular regeneration. It is worth noting that the proliferation and multi-directional differentiation potential of OFSCs decreased with age, so the therapeutic effect of OFSCs in elderly patients may be limited.^[35,36]^

In addition to their multipotent differentiation potential, orbital fat-derived stem cells have similar anti-inflammatory and immunosuppressive abilities to bone marrow mesenchymal stem cells. Ko-JoLin et al^[30]^ found that OFSCs have anti-inflammatory ability to inhibit macrophage aggregation and infiltration. Chen et al^[37]^ found that when OFSCs were mixed with hepatocytes from patients with acute liver failure, orbital septum adipose stem cells secreted a large amount of cytokine IL-6, which played an immunomodulatory and protective role in hepatocytes exposed to serum from patients with acute liver failure. Hsiao et al^[38]^ found that in animal models of testicular torsion, injection of OFSCs could prevent testicular torsion induced infertility and inhibit oxidative stress and apoptosis in testis. Lien et al^[39]^ pointed out that OFSCs could inhibit the proinflammatory ability and proliferation of macrophages through paracrine. In conclusion, OFSCs have anti-inflammatory and immunomodulatory abilities, so they have broad research prospects in the treatment of various diseases involving acute and chronic inflammatory injuries.

## 7. Prospect

Orbital fat has a special embryological origin, and the unique histologic structure may make it have some physiological functions different from subcutaneous fat. Fibroblasts and adipose stem cells derived from orbital fat may be the cytological basic components of specific physiological functions and pathological changes of orbital fat. The value of orbital adipose stem cells in the field of regenerative medicine such as eyes needs to be further studied. In addition, the anti-inflammatory and immunosuppressive abilities of OFSCs need to be further studied and explored in various diseases involving acute and chronic inflammation.

On the whole, the research of OFSCs in the field of corneal regeneration has made great progress, but the existing experimental methods are defected.^[33,34]^ Therefore, using OFSCs to create a more perfect corneal regeneration technology is a difficulty that must be overcome before clinical application. Although the histology and cytology of orbital fat have been discussed in detail in this paper, there are inevitably some limitations. For example, there is no sufficient research evidence to support the discussion of the special physiological function of orbital fat. At the same time, this paper mainly focuses on the English literature in recent years, and it may be possible to ignore some important literature.

## Author contributions

**Conceptualization:** Weicheng Gao.

**Writing – original draft:** Changhao Li.

**Writing – review & editing:** Shenzhen Gao.
